# Applying AI and Guidelines to Assist Medical Students in Recognizing Patients With Heart Failure: Protocol for a Randomized Trial

**DOI:** 10.2196/49842

**Published:** 2023-10-24

**Authors:** Hyeon Joo, Michael R Mathis, Marty Tam, Cornelius James, Peijin Han, Rajesh S Mangrulkar, Charles P Friedman, VG Vinod Vydiswaran

**Affiliations:** 1 Department of Learning Health Sciences University of Michigan Ann Arbor, MI United States; 2 Department of Anesthesiology University of Michigan Ann Arbor, MI United States; 3 Department of Internal Medicine Cardiology University of Michigan Ann Arbor, MI United States; 4 Department of Pediatrics University of Michigan Ann Arbor, MI United States; 5 Department of Internal Medicine University of Michigan Ann Arbor, MI United States; 6 Department of Computational Medicine and Bioinformatics University of Michigan Ann Arbor, MI United States; 7 School of Information University of Michigan Ann Arbor, MI United States

**Keywords:** medical education, clinical decision support systems, artificial intelligence, machine learning, heart failure, evidence-based medicine, guidelines, digital health interventions

## Abstract

**Background:**

The integration of artificial intelligence (AI) into clinical practice is transforming both clinical practice and medical education. AI-based systems aim to improve the efficacy of clinical tasks, enhancing diagnostic accuracy and tailoring treatment delivery. As it becomes increasingly prevalent in health care for high-quality patient care, it is critical for health care providers to use the systems responsibly to mitigate bias, ensure effective outcomes, and provide safe clinical practices. In this study, the clinical task is the identification of heart failure (HF) prior to surgery with the intention of enhancing clinical decision-making skills. HF is a common and severe disease, but detection remains challenging due to its subtle manifestation, often concurrent with other medical conditions, and the absence of a simple and effective diagnostic test. While advanced HF algorithms have been developed, the use of these AI-based systems to enhance clinical decision-making in medical education remains understudied.

**Objective:**

This research protocol is to demonstrate our study design, systematic procedures for selecting surgical cases from electronic health records, and interventions. The primary objective of this study is to measure the effectiveness of interventions aimed at improving HF recognition before surgery, the second objective is to evaluate the impact of inaccurate AI recommendations, and the third objective is to explore the relationship between the inclination to accept AI recommendations and their accuracy.

**Methods:**

Our study used a 3 × 2 factorial design (intervention type × order of prepost sets) for this randomized trial with medical students. The student participants are asked to complete a 30-minute e-learning module that includes key information about the intervention and a 5-question quiz, and a 60-minute review of 20 surgical cases to determine the presence of HF. To mitigate selection bias in the pre- and posttests, we adopted a feature-based systematic sampling procedure. From a pool of 703 expert-reviewed surgical cases, 20 were selected based on features such as case complexity, model performance, and positive and negative labels. This study comprises three interventions: (1) a direct AI-based recommendation with a predicted HF score, (2) an indirect AI-based recommendation gauged through the area under the curve metric, and (3) an HF guideline-based intervention.

**Results:**

As of July 2023, 62 of the enrolled medical students have fulfilled this study’s participation, including the completion of a short quiz and the review of 20 surgical cases. The subject enrollment commenced in August 2022 and will end in December 2023, with the goal of recruiting 75 medical students in years 3 and 4 with clinical experience.

**Conclusions:**

We demonstrated a study protocol for the randomized trial, measuring the effectiveness of interventions using AI and HF guidelines among medical students to enhance HF recognition in preoperative care with electronic health record data.

**International Registered Report Identifier (IRRID):**

DERR1-10.2196/49842

## Introduction

Computer-based diagnostic systems have played a critical role in both clinical practice and medical education, enhancing diagnostic accuracy and fostering the development of necessary knowledge and skills [1]. According to the Institute of Medicine’s report [2], the use of informatics for clinical decision-making is an essential educational competency required for all health care professionals. However, the informatics landscape has undergone rapid advancement with the advent of deep learning in artificial intelligence (AI) and has revolutionized disease diagnosis, treatment delivery, and patient care [3-5]. Subsequently, AI-based tools become increasingly prevalent in health care, allowing health care providers to make informed clinical decisions for high-quality care and optimal patient outcomes [6-8].

As AI continues to be integrated into medical practice, it becomes inevitable for medical students, residents, and professionals to acquire the necessary skills for effective medical practice [9]. In 2021, Lomis et al [10] highlighted the importance of incorporating AI training across health care professions to maximize its benefits while mitigating the potential drawbacks in routine patient care. Mckinsey’s report [11], titled “Transforming Healthcare with AI,” emphasizes the need to implement this transformation within the realm of education and training. Moreover, AI training within the medical school curriculum is an active area of discussion and investigation [10,12,13]. With AI in the medical program, students have the opportunity to learn about the use of apps and limitations of AI in clinical practice and improve their ability to use AI-based tools to enhance patient care and decision-making.

In 1989, Iliad [14,15], a computer-aided diagnosis, was introduced to enhance diagnostic abilities, encompassing over 6300 disease manifestations and addressing 1300 diseases related to internal medicine. Iliad offered a unique diagnostic learning experience by simulating cases and guiding users through a series of decision-making processes encountered in clinical workups. It also provided tailored feedback at each decision-making of the clinical workups based on the user’s performance. Lincoln et al [16] and Lange et al [17] demonstrated the improvement of diagnostic reasoning and a diagnostic error reduction among medical students and nurse practitioner students. Similarly, Friedman et al [18] confirmed enhanced diagnostic accuracy before and after using diagnostic consultation systems among medical students, residents, and physicians.

Despite the benefits of diagnostic systems, their integration into educational programs has been limited and has insufficient results [19,20]. Berner and McGowan [1] also pointed out that 1 contributing factor to limited educational usage is the stand-alone nature of these systems, which needs to be better integrated into existing clinical workflows. Conversely, Tolsgaard et al [21] observed that commercially available AI systems used in clinical settings are predominantly tailored to address specialized clinical challenges rather than assisting health care providers in skill enhancement. As a result, there is a gap in the development of health care professionals’ ability to use such decision support systems to use algorithmically generated recommendations in their clinical decision-making responsibly.

Moreover, an overreliance on AI systems can lead to unintended errors due to automation bias [22], which refers to the tendency for individuals to place excessive trust in automated systems and disregard their own judgment, such as the failure of autopilot technology [23]. Without proper training on the responsible use of AI, health care professionals may unknowingly commit mistakes by relying too heavily on AI-based systems [24,25]. The lack of understanding and awareness of AI’s limitations and potential biases can lead to errors in decision-making and patient care [26]. Health care professionals need the knowledge and skills to critically evaluate and interpret AI-generated recommendations, taking into account contextual factors and individual patient needs [27].

To mitigate the adverse effects and ensure the responsible use of AI, Tolsgaard et al [21] emphasized the importance of integrating learning sciences with clinical science and data science while developing new AI systems. This interdisciplinary approach aims to combine insights from educational research, clinical practice, and data analysis to design AI systems [28]. This approach enhances performance and fosters continuous learning and professional development. By integrating these disciplines, AI systems can be designed to provide appropriate feedback, facilitate reflective practice, and support the acquisition of clinical reasoning skills, promoting a balance between clinical practice and education in health care.

This paper is centered on demonstrating a study protocol, including a study design, a systematic procedure for case selection from electronic health record (EHR) data, and intervention designs to improve the recognition of patients with heart failure (HF) before surgery in a preoperative care clinical setting. HF is a common and serious chronic condition characterized by an abnormality in the structure and function of the heart, leading to reduced blood circulation throughout the body [29,30]. However, identifying patients with HF remains challenging due to its subtle and concurrent progression with other conditions and the lack of a single gold standard diagnostic test for HF [31,32]. Several algorithmic approaches have been recently published to improve HF detection, such as convolutional neural network with ECG [33-35], logistic regression [36], recurrent neural network [37], and transformer [38] models with EHR data.

The primary objective of this study is to measure the effectiveness of interventions aimed at improving HF recognition in preoperative care through a web-based clinical decision support (CDS) tool. The interventions are grounded in our previous research using traditional machine learning (ML) algorithms [39] and guideline-based diagnostic factors reviewed by clinical experts [40,41]. These interventions are algorithmically and manually synthesized from a large amount of EHR data for identifying HF. The secondary objective is to evaluate the impact of inaccurate ML recommendations in recognizing patients with HF, as compared to HF guideline-based recommendations. The third objective is to explore the relationship between the inclination to accept AI recommendations and the ability to accurately recognize HF using ML-based interventions.

## Methods

### Study Setting and Participants

Participants in this study are medical students to simulate the clinical task of preoperative surgical screening, targeting the recognition of HF as a specific educational objective using surgical cases performed at a tertiary hospital. This study’s participants review surgical cases on our internally developed web-based CDS educational tool that displays deidentified EHR data, including clinical visits, diagnoses, procedures, vitals, laboratory, medications, imaging or studies, and clinical notes up to the day of surgery. To maximize the efficiency of case reviews, clinical data included in the CDS tool was tailored to preoperative care and cardiovascular diseases for screening HF. Irrelevant EHR data to this study (eg, administrative data, non-HF-related imaging) was excluded. This CDS tool was developed and deployed in a secure internal environment where study participants can access and review surgical cases.

The eligible students for participation in this study are medical students in their third (M3) or fourth (M4) year of medical school, who completed some coursework requirements and underwent clinical rotations. In addition, medical school graduates who have not yet started their residency program are eligible for inclusion. The rationale behind including senior medical students in this study is that accurate identification of HF using real-world EHR data needs a comprehensive understanding of clinical knowledge and skills acquired through coursework and clinical rotations.

This study is entirely on the internet and comprises two components as follows:

1. The first component entails an e-learning module, including an instructional video about the intervention and a short quiz, with an estimated completion time of 30 minutes.
2. The second component involves reviewing 20 surgical cases to evaluate the presence or absence of HF using EHR data, with an estimated completion time of 90 minutes.

Prior to reviewing the surgical cases, participants are required to complete a 5-question short quiz with all correct answers. They have the option to attempt the quiz multiple times if necessary. As this study is conducted on the internet, participants can complete this study at their own pace and at their own convenient time. Upon successful completion of this study, participants receive US $50 as a gift of appreciation.

### Ethics Approval

This research study involving human subjects has received ethical approval from the institutional review board at the University of Michigan (HUM00207646). This approval indicates that this study’s protocol, including research design, data collection methods, and informed consent, has been reviewed and considered to be in compliance with ethical standards for human subject research.

### Study Design

The study design of this HF recognition study is a 3 × 2 factorial randomized trial that involves 3 intervention groups and the order exchange of 2 sets of surgical cases during the pre- and posttests. This study design allows us to measure the effectiveness of 3 interventions without introducing bias from surgical case selection in the pre- and posttests, summarized in Multimedia Appendix 1.

The rationale behind exchanging the order of 2 sets is to minimize the potential bias arising from differences in individual surgical cases. For example, if set A in the pretest contains 10 surgical cases that are more difficult than those in set B in the posttest, it would be challenging to attribute any improvements in accuracy solely to the interventions. Therefore, exchanging the order of the sets between the pre- and posttests ensures the measurement of unbiased interventions’ effectiveness.

Furthermore, the use of random selection for the surgical cases is not suitable in this study due to the implementation of specific case selection criteria. The selection criteria aim to provide medical students with targeted learning opportunities to recognize HF through the review of surgical cases. The selection process is designed to ensure that the chosen cases offer valuable educational experiences and align with the intended learning objectives. Further details about the case selection are discussed in the later section dedicated to the systematic procedure for surgical case selection.

Prior to implementing the 3 × 2 factorial study design, a pilot study was conducted using a 3-arm study design. In this preliminary investigation, participants were asked to determine the presence or absence of HF before intervention access and then reassess the same surgical case after intervention access. However, this study design demonstrated an “anchoring effect,” where participants tended to adhere to their initial clinical judgment. Therefore, it underestimated the effectiveness of the intervention. Consequently, the decision was made to adopt the 3 × 2 factorial design to overcome this limitation and provide a more accurate evaluation of the effectiveness of interventions.

### Interventions: ML_DR_, ML_IR_, and EB References

In order to measure the effectiveness of AI and guideline-based educational methods with 3 distinct interventions, the interventions need to provide essential information specific to the methods to enhance the ability to accurately recognize HF using patient data from the EHR.

The essential information is consolidated into a single *reference* page, which compiles risk factors associated with HF and method-specific recommendations (eg, HF yes or no, risk scores) to augment medical students’ ability to recognize HF accurately. The risk factors are categorized into nine sections based on consultations with HF experts to improve the readability and comprehensibility of the interventions. These sections include (1) signs and symptoms, (2) past HF history, (3) past medical history, (4) surgical history, (5) medications, (6) physical exams, (7) test labs, (8) imaging or studies, and (9) ECG. A detailed description of the risk factors within each section can be found in Multimedia Appendix 2.

**Figure 1:**
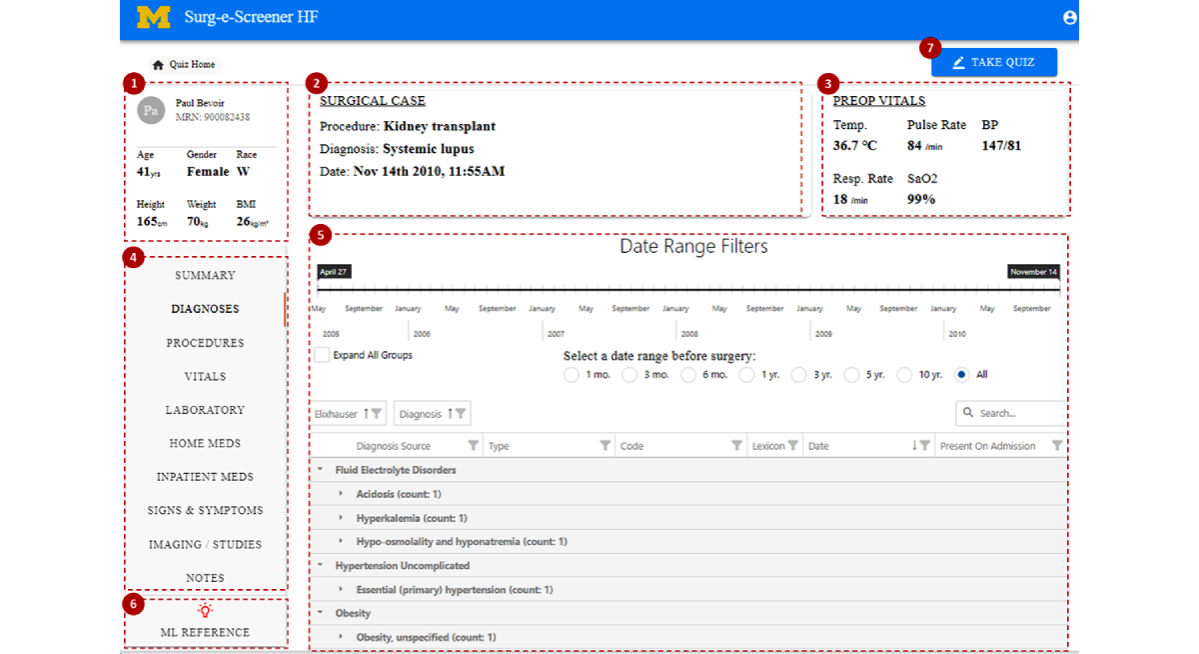
A web-based educational tool to review surgical cases, including (1) demographics (top left), (2) surgical case information (top middle), (3) preoperative vitals (top right), (4) subject domains (left side), (5) EHR data of a subject domain (center), (6) ML and EB intervention (left bottom). The intervention tab is only available during the posttest. After reviewing surgical cases, answer survey questions by clicking the (7) “TAKE QUIZ” button. EB: evidence based; EHR: electronic health record; ML: machine learning.

During the posttest phase, the intervention, represented by the reference page, is available to medical students who completed the first 10 surgical cases using only their clinical judgment during the pretest. In the posttest, the reference page is accessible through an ML or EB reference tab (6) in Figure 1 and can be accessed at any time while medical students review a surgical case. Further information about developing the ML model used in ML reference is included in Multimedia Appendix 3, and the details of the intervention differences are outlined as follows.

#### ML_DR_ Reference

The ML_DR_ reference provides a direct recommendation (DR), which is an output from the ML algorithm in the form of the presence or absence of HF, a dichotomous response. To achieve this, an optimal threshold is set to discriminate between positive and negative cases based on the estimated HF probability. The threshold configuration is critical in determining the sensitivity and specificity of the system. In this study, the optimal threshold was determined to be 38% based on the assumption that sensitivity and specificity are equally important. The ML_DR_ reference includes a brief explanation of the dichotomous recommendation, such as “this patient has a 98% chance of having HF, which is above the 38% threshold,” to allow medical students to incorporate this into their clinical decision-making. It is important to note that the diagnosis of HF is a complex process that requires a thorough examination, and ML_DR_ reference offers a simple and efficient method to recommend the presence or absence of HF to medical students, along with a justification of the recommendation. The screenshot of ML_DR_ reference is in Multimedia Appendix 4.

#### ML_IR_ Reference

ML_IR_ reference offers an indirect recommendation (IR), which is a proxy of carrying HF risk, using the area under the receiver operating characteristic (AUROC), which comprises the true-positive rate (TPR) and false-positive rate (FPR). The AUROC plot, with TPR and FPR plotted along its axes, provides a comprehensive view of the model’s performance across various thresholds. In contrast to ML_DR_ reference, which is limited to a single threshold, ML_IR_ reference shows the position of TPR and FPR on the AUROC plot when the threshold is defined as the predicted probability of HF for individual cases. For example, in the case of a surgical case with a high predicted HF probability, ML_IR_ reference displays the corresponding TPR or FPR position on the AUROC plot, reflecting a high-level threshold equivalent to the predicted HF probability. This approach enables medical students to examine surgical cases with different TPR and FPR positions on the AUROC plot, thereby avoiding a *decision bias* toward a single threshold. The screenshot of ML_IR_ reference is in Multimedia Appendix 5. In short, ML_DR_ and ML_IR_ references adopt 2 distinct approaches to present direct and indirect recommendations to medical students while using the same underlying ML model.

#### EB Reference

The third intervention, *EB reference,* includes risk factors described in HF guidelines [42,43] and shows the presence or absence of these risk factors explicitly documented in EHR and clinical experts’ impressions while reviewing the case from EHR. In our previous study [40], the 20 surgical cases were adjudicated by a consensus panel of HF experts (cardiologists, cardiac anesthesiologists, and critical care physicians) who indicated the explicit documentation of the risk factors in EHR as well as their impression of presence or absence of the risk factors. Unlike the risk factors from EHR data in ML references, this intervention uses the highest-quality evidence in the medical literature from various clinical studies and a consensus of domain experts to incorporate evidence-based practice into HF diagnostic recommendations. Further, because the HF recommendations in the guidelines [42,43] are intended to provide evidence-based recommendations for health care practitioners, they are presented in a way that is easy to *understand* and *acceptable* for clinicians. As such, the risk factors related to heart diseases are incorporated into medical education and training, resulting in less friction in clinical reasoning and decision-making. The screenshot of the EB reference is in Multimedia Appendix 6.

**Figure 2:**
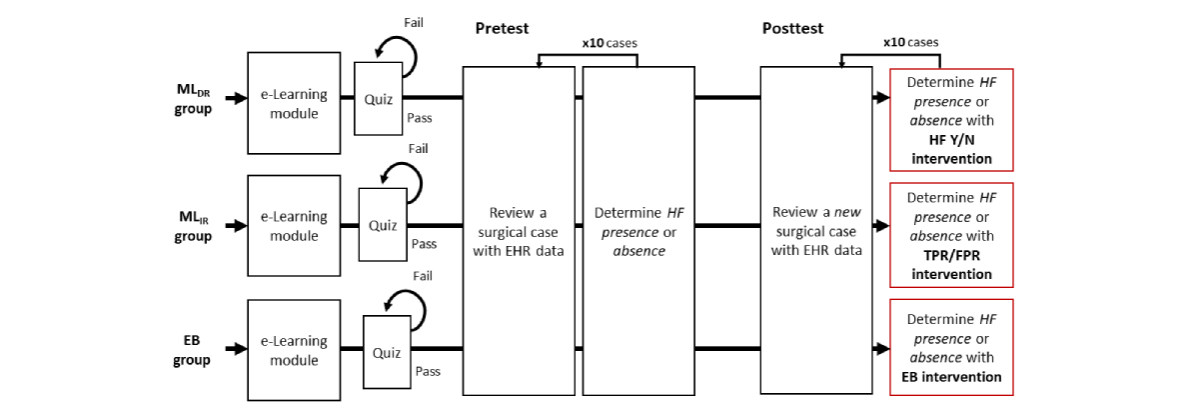
An overview of study participation using a 3 × 2 factorial study design. The participation includes an e-learning module, a short quiz, and 20 surgical case reviews. The order of 2 sets of 10 surgical cases is predetermined before the participant begins this study’s activities. ML_DR_ group: a group of students who receive ML-generated risk factors and a direct recommendation of HF Y/N. ML_IR_ group: a group of students who receive ML-generated risk factors and an indirect recommendation of the likelihood of having HF from TPR, FPR, and AUROC. EB group: a group of students who receive HF expert-reviewed risk factors extracted from HF guidelines. AUROC: area under the receiver operating characteristic; EB: evidence based; EHR: electronic health record; FPR: false-positive rate; HF: heart failure; ML: machine learning; TPR: true-positive rate; Y/N: yes or no.

Each group in the HF recognition study is given access to one of the interventions during the posttest case reviews. After assignment to an intervention group, all participants reviewed the same 20 surgical cases, 10 surgical cases during the pretest phase, and then 10 new surgical cases during the posttest phase. The intervention is only accessible during the posttest. Figure 2 shows an overview of this study’s participants for the three intervention groups: (1) ML_DR_ group, which receives direction recommendations indicating the presence or absence of HF; (2) ML_IR_ group, which receives indirect recommendations in the form of TPR, FPR, and AUROC plot; and (3) EB group, which receives expert-reviewed evidence-based risk factors from HF guidelines.

### E-Learning Module

Prior to starting the surgical case review, participants are required to engage in a series of preparatory activities, including a web-based e-learning module that includes a 20-minute prerecorded instructional video, and a short quiz with 5 multiple-choice questions. The purpose of the e-learning module is to provide participants with the necessary knowledge and understanding to recognize patients with HF using the web-based tool. Given the diversity of participants’ backgrounds and lack of familiarity with the new tool, this module offers educational materials and essential concepts that are fundamental to comprehending the interventions used to facilitate clinical decision-making.

The e-learning module focuses explicitly on facilitating participants’ comprehension of machine learning concepts, including predicted risk scores, important features, thresholds, and evaluation metrics (eg, sensitivity, specificity, TPR, and FPR) for ML intervention groups. Additionally, the module covers the identification of risk factors contributing to HF, as outlined in the American Heart Association [42,44] and the European Society of Cardiology [43] HF guidelines, and Framingham criteria [45] for the EB intervention group.

In an attempt to confirm that subjects have viewed and comprehended the material presented in the prerecorded video, participants are required to complete a short quiz, which must be completed with a score of 100% (5 out of 5 questions). When questions are answered incorrectly, participants are instructed to review the material and revise their responses prior to proceeding to the next step. This e-learning module reduces variations in participants’ prior knowledge and background to develop a standardized basis for evaluating surgical cases and measuring the effectiveness of interventions.

### Surgical Case Selections

In our previous study [40,41], a consensus panel of HF experts, comprising cardiologists, cardiac anesthesiologists, and critical care physicians, assessed 1018 surgical cases, of which 703 were available during this study. These cases were part of a stratified subsample of 40,659 adult noncardiac surgical procedures between 2015 and 2019 at Michigan Medicine. Using a feature-based sampling process [46], 20 surgical cases were deliberately selected, considering factors, such as case complexity, expert consensus, ML performance, and HF outcomes. The 20 cases were divided into 2 sets, each consisting of 10 cases, for the use of pre- and posttests. Each set included an equal number of easy and difficult cases, as well as an equal representation of patients with and without HF.

**Figure 3:**
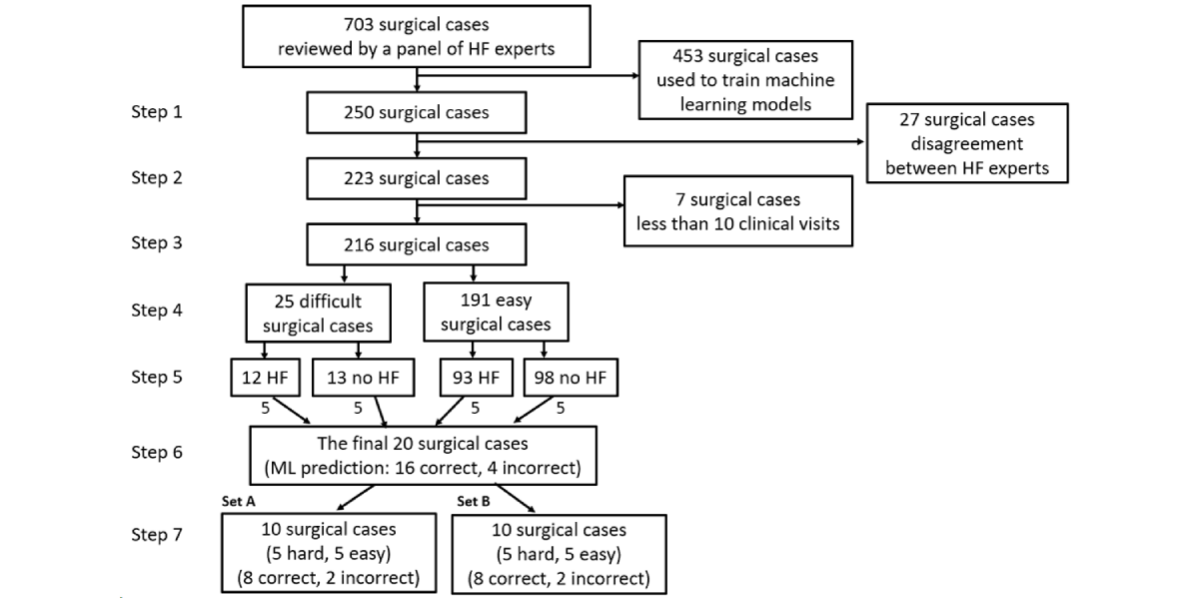
A systematic sampling procedure for the final 20 surgical cases with respect to the level of difficulty, positive and negative HF cases, and the ML performance from expert-reviewed surgical cases. HF: heart failure; ML: machine learning.

The systematic sampling procedure for surgical case selection is illustrated in Figure 3. There are seven steps involved: (1) selecting cases from the holdout data set, (2) including cases that are consensus among expert reviewers, (3) including patients who had at least 10 clinical visits, (4) determining the level of difficulty from clinical diagnostic factors presented in EHR, (5) selection priority based on HF outcome, (6) consideration of ML performance (90% AUROC, 82% sensitivity, and 82% of specificity in our model), and (7) dividing cases into two sets for pre- and posttests. The more detailed descriptions of each step are included in Multimedia Appendix 7.

The implementation of a systematic methodology for case selection from EHR is critical, as the validation derived from this study could serve as a foundational cornerstone for scalability across diverse medical conditions. For example, diagnostic tasks using ML with expert-reviewed labels are potential candidates that could be integrated into this CDS tool by following the steps above, requiring only minor adjustments to accommodate unique clinical nuances. Thus, these ML-based tasks could be transformed into additional educational resources and presented to learners through this educational tool.

In the last step, surgical cases in set A and set B were presented to medical students in pre- and posttests to determine the presence or absence of HF. However, it does not guarantee equivalent difficulty levels, which is critical for evaluating intervention effectiveness. To mitigate the issue, we altered the order of sets in the pre- and posttests. Half of the participants received set A in the pretest and set B in the posttest, and vice versa for the rest of the participants, resulting in a 3 × 2 factorial design that ensured a balanced measurement of the interventions’ effectiveness.

### Sample Size and Recruitment Strategies

To assess the effectiveness of interventions with a power of 80% at a significant level of 5%, the total number of subjects needed is 75, with 25 subjects assigned to each group. This power analysis was conducted using a 1-sample *t* test using SPSS (version 28.0; IBM Corp), a statistical software package. The power analysis was based on our preliminary results, which indicated a mean difference of 0.08 and an SD of 0.13.

The recruitment for this study commenced in August 2022 and will continue until December 2023. The primary recruitment method involves sending a group email to year 3 and 4 medical students through faculty and academic curriculum coordinators, posting notices in various institutional newsletters and web-based bulletin boards, and distributing fliers. The recruitment started at a single institution and expanded to participants from other institutions.

Furthermore, snowball sampling has been used, which asks for referrals from enrolled study participants. To encourage referrals, a referral-based lottery has been implemented for individuals who refer their friends and colleagues in year 3 or 4 medical students. This lottery is recurring quarterly, and the winner will be drawn each term and awarded a US $100 prize. In addition, we have implemented a sweepstakes for study participants who will enter for a chance to win US $500 worth of prizes to recruit more students.

### Assignment of Interventions

To participate in this study, subjects must first complete a survey form providing information about their school year, clinical experiences, and their level of acceptance toward clinical recommendations made by both clinical experts and AI algorithms. As an individual’s behavior and willingness to accept recommendations can significantly impact this study’s outcome [47], the level of acceptance was included in the survey form for use in the subject assignment.

Upon registration, subjects are allocated to one of the intervention groups using a permuted block and stratification method [48]. This method involves stratifying subjects based on key characteristics and randomly assigning them across groups within a fixed size of blocks. For this study, the acceptance level of AI recommendations (≥50%) and medical institutions serve as the stratification criteria, with subjects being randomly assigned to intervention groups within each block of size 6. As a result, each block consists of 2 occurrences of 3 distinct interventions.

### Data Collection

Upon completing the e-learning module, medical students begin reviewing 20 surgical cases. To assess the accuracy of recognizing patients with HF, medical students respond to a set of 3 survey questions for each case review. This survey is embedded in the web-based tool, which is accessible after reviewing EHR data and intervention information. Medical students have the option to revisit data points if needed to address the survey questions. As shown in Multimedia Appendix 8, 3 survey questions are presented to evaluate the ability of medical students to accurately identify the presence or absence of HF. The first question pertains to the final decision of HF presence or absence at the start of surgery, followed by a question on their level of confidence in their clinical decision. Finally, medical students are requested to check the type of information they use to inform their clinical decision-making process.

### Outcomes

The primary outcome of this study is to assess the effectiveness of interventions in improving the accuracy of recognizing patients with HF, by measuring the mean difference of recognition accuracy before and after intervention in pre- and posttests. Specifically, this study will demonstrate the mean difference in HF recognition accuracy before and after accessing ML_DR_ ML_IR_, and EB reference interventions.

The second outcome of this study is to evaluate the impact of accurate or inaccurate ML recommendations on medical students’ clinical judgment to recognize the presence or absence of HF, compared to the EB intervention group. In addition, this study explores the differential effects of direct and indirect ML recommendations on clinical decision-making among students.

Lastly, this study measures the effect of the acceptance level of AI reported in the prescreening survey on the accuracy of recognizing HF using ML references. This exploratory outcome will provide valuable insights for enhancing the acceptability of AI tools.

## Results

As of July 2023, 62 of the enrolled medical students have fulfilled this study’s participation, including the completion of a short quiz and the review of 20 surgical cases. The recruitment process for these study participants began in August 2022 and will end in December 2023.

## Discussion

### Principal Findings

In this study protocol paper, we outlined a methodological approach for measuring the effectiveness of ML-based interventions, both direct and indirect recommendations, and EB intervention. The interventions were integrated with EHR data within a web-based educational tool to enhance the recognition of HF before surgery. Specifically, we demonstrated a novel randomized trial using a 3 × 2 factorial design, systematic procedures for surgical case selection, and 3 interventions using ML algorithms and HF guidelines using EHR data.

The demonstration of 3 × 2 factorial design (intervention type × order of prepost sets) represents a unique methodological contribution, enabling the measurement of intervention effectiveness through enrolling students in pre- and posttests. Prior work such as Lincoln et al [16] with medical students and Lange et al [17] with nurse practitioner students adopted a 2 × 2 × 2 mixed factorial design (disease sets × trained or untrained groups × replication) to evaluate diagnostic errors and posterior probability pertaining to the comprehensiveness of clinical workups. In contrast to Iliad, which offers feedback during training via simulated cases, our study provides an e-learning module designed to impart and apply synthesized information, grounded in AI or HF guidelines, to enhance diagnostic accuracy. With our proposed factorial design, the effectiveness of the synthesized information was measured before and after reviewing surgical cases derived from EHR.

Furthermore, this study protocol paper articulates a systematic procedure of selecting HF cases from expert-reviewed surgical cases in EHR, incorporating the difficulty level and positive and negative outcomes. We believe that this systematic procedure of case selection not only mitigates selection bias for this study but also offers a replicable framework for other diseases when expert-reviewed labels and EHR data are available. In addition, our approach has the potential of scalability to present algorithmically or manually synthesized information, along with other data available in EHRs, on other diseases. This is challenging for expert systems like Iliad, equipped with a knowledge base or inference engine [14,49].

### Limitations

While this study provides a foundational approach to measure the effectiveness of enhancing HF recognition, it is important to note limitations with respect to a limited scope of findings and generalizability. Specifically, the target sample size of 75 participants (25 in each study arm) is adequate with a statistical power of 0.80 to measure the effectiveness of enhancing HF decision-making before and after intervention access. However, additional research questions, such as comparative effectiveness between ML and EB interventions, are limited in this study. Furthermore, this study was primarily conducted within an academic medical center that could be limited to a representative of medical schools in the United States. Despite this sample size constraint, our proposed study has the potential to inform future studies to broaden our understanding of how AI-based tools can be helpful for medical education and training.

Moreover, this study primarily focuses on intervention effectiveness, neglecting the essential facet of learner engagement and feedback mechanisms within the web-based tool. To enhance the effective learning outcomes in future iterations, the web-based decision support tool could incorporate real-time feedback upon each case completion, such as indicating the accuracy of case reviews and providing preannotated expert commentary on examined surgical cases. Additionally, offering structured guidance on interpreting AI-generated outputs is critical for accurately using predictive scores and in-depth analysis of misclassification instances. Integrating these postreview feedback mechanisms holds considerable promise for aiding learners in effectively leveraging AI-based tools for clinical decision-making.

The ML model used in this study was trained and evaluated on a stratified and manually reviewed data set, comprising patients who had developed HF and patients at high and low risk [40,41]. While stratification ensures the model learns various stages of HF progression, it may not accurately reflect the prevalence of HF estimated between 1.5% and 1.9% in the US population [50]. As a result, the model performance should be interpreted with caution when considering its use in a routine clinical setting and requires careful further examination. In this study, however, the deliberate inclusion of surgical cases for training the model with various complexities serves educational purposes effectively. This approach allows learners to gain experience in clear-cut positive or negative outcomes and also borderline cases, which are inherently challenging yet particularly informative.

### Conclusions

In summary, this study protocol demonstrates a study design for measuring the effectiveness of interventions to enhance HF recognition among adult noncardiac surgical patients. Despite limitations of sample size and generalizability, this study serves as a foundational step toward a more comprehensive understanding of how AI techniques and evidence-based medicine can be synergistically used to advance both medical education and patient care outcomes.

## Appendix

Multimedia Appendix 1 A 3 x 2 factorial study design with three interventions and the set order of surgical cases. A study subject reviews a total of 20 surgical cases, 10 surgical cases in each pre- and post-tests. The mean difference in accuracy before and after accessing an intervention will be measured in each cell.

Multimedia Appendix 2 ML and EB Reference tables. The nine sections of heart failure–related risk indicators are categorized in the sequence of informed necessity for diagnosis.

Multimedia Appendix 3 A summary of developing and adopting a machine learning model in ML References.

Multimedia Appendix 4 ML_DR_ Reference: This intervention includes a direct recommendation based on heart failure (HF) probability, like HF or No HF. It also consists of the top 5 risk factors and the entire list in the reference table.

Multimedia Appendix 5 ML_IR_ Reference: This intervention includes the true-positive rate, false-positive rate, and area under the receiver operating characteristic plot. These will give a proxy of heart failure risk level but no direct recommendation. It also consists of the top 5 risk factors and the entire list in the reference table.

Multimedia Appendix 6 EB Reference: This intervention includes heart failure (HF) expert-reviewed presence or absence of risk factors listed in HF guidelines and HF expert’s impression on the presence or absence of risk factors as reviewing a surgical case.

Multimedia Appendix 7 This is a detailed 7-step procedure to systematically select two sets of surgical cases from electronic health records taking into account factors such as case complexity, expert agreement, machine learning performance, and heart failure outcome.

Multimedia Appendix 8 The questionnaire that study participants are asked to answer after reviewing each case via the web-based educational tool.

Multimedia Appendix 9 The disclosure of the use of ChatGPT or other generative language models.

## Abbreviations

AI: artificial intelligence
AUROC: area under the receiver operating characteristic
CDS: clinical decision support
EHR: electronic health record
FPR: false-positive rate
HF: heart failure
ML: machine learning
TPR: true-positive rate

## Data Availability

Due to patient identification concerns and confidentiality, the data used for this study is not publicly available. Any request for data access may need additional approval from the institute review board at Michigan Medicine.
