# Supplementary material for: Applying AI and Guidelines to Assist Medical Students in Recognizing Patients With Heart Failure: Protocol for a Randomized Trial
Source: JMIR Res Protoc. 2023 Oct 24;12:e49842. doi: 10.2196/49842 (PMC10630872; doi:10.2196/49842)
Supplement: Multimedia Appendix 1 [file resprot_v12i1e49842_app1.docx]

**Multimedia Appendix 1.** A 3x2 factorial study design with three interventions and the set order of surgical cases. A study subject reviews a total of 20 surgical cases, 10 surgical cases in each pre- and post-tests. The mean difference in accuracy before and after accessing an intervention will be measured in each cell.

|  | **The order of surgical case sets in pre- and post-tests** | |
| --- | --- | --- |
|  | **Set A (Pre) -> Set B (Post)** | **Set B (Pre) -> Set A (Post)** |
| **ML_DR_ Group** | Mean (post – pre) | Mean (post – pre) |
| **ML_IR_ Group** | Mean (post – pre) | Mean (post – pre) |
| **EB Group** | Mean (post – pre) | Mean (post – pre) |

**ML_DR_ Group**: a group of students who receive risk factors and a direct recommendation of HF Y/N generated by an ML algorithm.

**ML_IR_ Group**: a group of students who receive risk factors and an indirect recommendation of the likelihood of having HF from TPR, FPR, and AUROC, generated by an ML algorithm.

**EB Group**: a group of students who receive HF expert-reviewed risk factors extracted from HF guidelines
