# Supplementary material for: Applying AI and Guidelines to Assist Medical Students in Recognizing Patients With Heart Failure: Protocol for a Randomized Trial
Source: JMIR Res Protoc. 2023 Oct 24;12:e49842. doi: 10.2196/49842 (PMC10630872; doi:10.2196/49842)
Supplement: Multimedia Appendix 2 [file resprot_v12i1e49842_app2.docx]

**Multimedia Appendix 2.** ML and EB Reference tables. The nine sections of HF-related risk indicators are categorized in the sequence of informed necessity for diagnosis.

| **Evidence-Based Reference Table** |  |
| --- | --- |
| Category | HF Risk Factors |
| Signs and Symptoms | Dyspnea On Exertion |
|  | Nocturnal Cough |
|  | Paroxysmal Nocturnal Dyspnea Orthopnea |
| Past Medical History | Hypertension |
|  | Coronary artery disease (CAD) History |
| Medications | Diuretics |
|  | Cardiotoxic |
| Physical Exam | Pulmonary Rales |
|  | Displaced Apical Beat |
|  | Hepatojugular Reflux |
|  | Heart Murmur |
|  | Hepatomegaly |
|  | WeightLoss |
|  | Neck Vein Distension |
|  | Tachycardia |
|  | Third Heart Sound |
|  | Ankle Edema |
| Test - Lab | NT-proB-type Natriuretic Peptide (NT-pro BNP)  (BNP) |
| Test - Image | Pleural Effusion |
|  | Acute Pulmonary Edema |
|  | Ascites |
|  | LVEF value (1yr before surgery) |
|  | LVEF value (Anytime before surgery) |
|  | Cardiomegaly |
| Test - ECG | ECG Abnormality |

| **Machine Learning Reference Table** | |  |
| --- | --- | --- |
| Category | Feature Name | Percentage (%) |
| Signs and Symptoms | abdominal discomfort* | -0.40 |
|  | Acute Pain* | 0.75 |
|  | Arthralgia* | 0.31 |
|  | calf pain* | -0.73 |
|  | chest discomfort* | 1.47 |
|  | chronic low back pain* | -0.65 |
|  | cramping* | -0.68 |
|  | diffuse abdominal pain* | 1.19 |
|  | dry skin* | 1.26 |
|  | dyspnea at rest* | 0.58 |
|  | dyspnea on exertion* | 1.00 |
|  | eye pain* | 0.93 |
|  | fatigue* | 1.12 |
|  | Flushed* | -0.68 |
|  | Heartburn* | 0.99 |
|  | lightheadedness* | 0.42 |
|  | low back pain* | -0.36 |
|  | neck and back pain* | -0.87 |
|  | photophobia* | 1.36 |
|  | rashes* | 1.07 |
|  | rebound tenderness* | -0.35 |
|  | respiratory distress* | 1.01 |
|  | Snoring* | -0.40 |
|  | stridor* | 0.78 |
|  | Syncope* | 1.13 |
|  | urinary symptoms* | 1.08 |
|  | visual hallucinations* | 1.06 |
|  | weakness* | 0.36 |
|  | wheezing* | 2.02 |
| Past HF History | Cardiomyopathy | 2.98 |
|  | Heart Failure | 4.32 |
| Past Medical History | Abnormalities of breathing | 1.68 |
|  | Age In Years | 0.65 |
|  | apnea* | -0.41 |
|  | Cardiac dysrhythmias | 1.66 |
|  | Comorbidity Diabetes Complicated | 1.30 |
|  | Comorbidity Hypertension Complicated | 1.40 |
|  | Comorbidity Hypertension Uncomplicated | 1.79 |
|  | Comorbidity Peripheral Vascular Disorders | 2.26 |
|  | Comorbidity Valvular Disease | 1.57 |
|  | Coronary Artery Disease | 2.10 |
|  | Elevated blood glucose level | 0.93 |
|  | Encounter for other and unspecified procedures and aftercare | 0.67 |
|  | Encounter for other special examination without complaint, suspected or reported diagnosis | 0.71 |
|  | Gastro-esophageal reflux disease | -0.85 |
|  | Other and unspecified anemias | 0.96 |
|  | Other and unspecified osteoarthritis | -1.58 |
|  | Other anxiety disorders | -0.90 |
|  | Other disorders of arteries and arterioles | 1.56 |
|  | Other disorders of fluid, electrolyte and acid-base balance | -0.44 |
|  | Other joint disorder, not elsewhere classified | 0.37 |
|  | Personal history of malignant neoplasm | -1.12 |
|  | regurgitation* | 2.26 |
| Past Surgical History | Insert artery catheter thru skin | 1.75 |
|  | Presence of cardiac and vascular implants and grafts | 3.38 |
|  | Under Intravenous Vascular Introduction and Injection Procedures | 0.53 |
| Medications | ANTIDOTES | -0.56 |
|  | AUTONOMIC DRUGS | 0.58 |
|  | BLOOD | -0.85 |
|  | CARDIAC DRUGS | 0.69 |
|  | DIAGNOSTIC | 0.13 |
|  | MUSCLE RELAXANTS | -1.45 |
|  | PSYCHOTHERAPEUTIC DRUGS | -0.27 |
|  | SEDATIVE/HYPNOTICS | 0.67 |
|  | SKIN PREPS | 1.24 |
| Physical Exam | Preop Blood Pressure Diastolic | 0.85 |
|  | Preop Blood Pressure Systolic | -0.82 |
|  | BMI max | 0.71 |
|  | BMI mean | 1.08 |
|  | BP Diastolic max | 0.40 |
|  | BP Diastolic var | -0.25 |
|  | BP Systolic max | -0.03 |
|  | BP Systolic var | -0.14 |
|  | Heart Rate max | 0.52 |
|  | Heart Rate min | 0.76 |
|  | Heart Rate var | -0.19 |
|  | Preop Pulse Rate | 0.96 |
|  | Preop Respirate Rate | 1.12 |
|  | Preop Temperature | -0.41 |
|  | pitting edema* | 1.25 |
|  | Respiratory Rate max | -0.61 |
|  | Preop SaO2 | -0.14 |
|  | SPO2 max | 0.10 |
|  | SPO2 min | -0.45 |
|  | Temperature max | -0.45 |
|  | Temperature mean | -0.56 |
|  | Temperature min | -0.43 |
| Test - Lab | Albumin last | -1.16 |
|  | BNP last | 0.72 |
|  | Creatinine last | 0.19 |
|  | HbA1c last | 0.86 |
|  | Hematocrit last | -0.11 |
|  | Hematocrit var | -1.30 |
|  | INR max | 0.07 |
|  | PlateletCount last | -1.54 |
|  | Preop EGFR | -0.77 |
|  | Sodium last | -0.77 |
|  | Sodium max | 0.03 |
|  | Sodium mean | -0.51 |
|  | Sodium min | -0.34 |
|  | Troponin last | -0.79 |
|  | Troponin max | -0.22 |
|  | WBC last | 1.41 |
|  | WBC max | -0.81 |
| Test - Image | LVEF min | -1.59 |
|  | LVEF var | -1.12 |
| Test - ECG | Atrioventricular and left bundle-branch block | 1.86 |
