# Supplementary material for: Applying AI and Guidelines to Assist Medical Students in Recognizing Patients With Heart Failure: Protocol for a Randomized Trial
Source: JMIR Res Protoc. 2023 Oct 24;12:e49842. doi: 10.2196/49842 (PMC10630872; doi:10.2196/49842)
Supplement: Multimedia Appendix 3 [file resprot_v12i1e49842_app3.docx]

**Appendix 3.** A summary of developing and adopting a machine learning model in ML References.

In our prior research, we assessed the performance of logistic regression, random forest, and extreme gradient boosting algorithms for predicting undiagnosed HF using ICD9 code-based labels. [39] Since 1,018 surgical cases were reviewed by a panel of HF experts who determined whether the patient carried HF at the start of the surgery [40,41], we refreshed the models with high-quality, expert-reviewed labels. The model training and evaluation from our previous work were followed, involving an 80-20 data split, cross-validation, and grid search for hyperparameter optimization, and the best model was evaluated on the test set.

The final model adopted in this study was logistic regression because of the model’s explainability. The coefficients of features can be translated into odds ratios, which offer valuable meaning about risk factors contributing to HF outcomes. The performance of the model on the test dataset was AUROC = 0.8998, Accuracy = 0.8223, Sensitivity = 0.8227, and Specificity = 0.8219. We utilized the scikit-learn library for machine learning tasks in Python. The sample code was published in our prior work publicly from Open Science Framework at <https://osf.io/kqj4f/> and further details are available in [39].
