# Supplementary material for: Applying AI and Guidelines to Assist Medical Students in Recognizing Patients With Heart Failure: Protocol for a Randomized Trial
Source: JMIR Res Protoc. 2023 Oct 24;12:e49842. doi: 10.2196/49842 (PMC10630872; doi:10.2196/49842)
Supplement: Multimedia Appendix 4 [file resprot_v12i1e49842_app4.docx]

**Multimedia Appendix 4.** ML_DR_ Reference: This intervention includes a direct recommendation based on HF probability, like HF or No HF. It also consists of the top 5 risk factors and the entire list in the reference table.

**
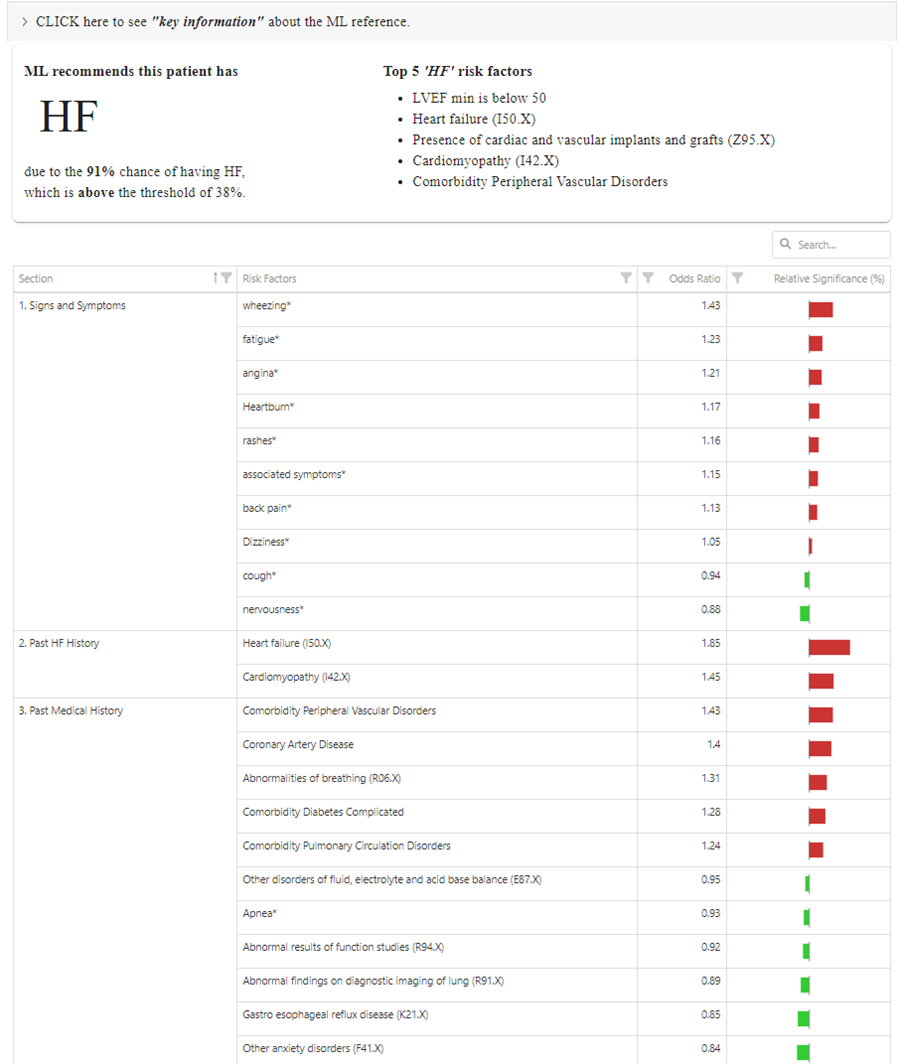
**
