# Supplementary material for: Applying AI and Guidelines to Assist Medical Students in Recognizing Patients With Heart Failure: Protocol for a Randomized Trial
Source: JMIR Res Protoc. 2023 Oct 24;12:e49842. doi: 10.2196/49842 (PMC10630872; doi:10.2196/49842)
Supplement: Multimedia Appendix 5 [file resprot_v12i1e49842_app5.docx]

**Multimedia Appendix 5.** ML_IR_ Reference: This intervention includes TPR, FPR, and AUROC plot. These will give a proxy of HF risk level but no direct recommendation. It also consists of the top 5 risk factors and the entire list in the reference table.

**
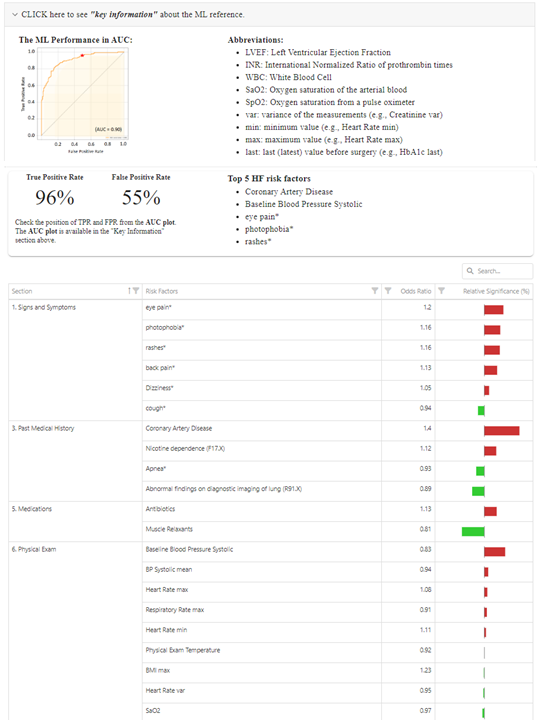
**
