# Supplementary material for: Applying AI and Guidelines to Assist Medical Students in Recognizing Patients With Heart Failure: Protocol for a Randomized Trial
Source: JMIR Res Protoc. 2023 Oct 24;12:e49842. doi: 10.2196/49842 (PMC10630872; doi:10.2196/49842)
Supplement: Multimedia Appendix 6 [file resprot_v12i1e49842_app6.docx]

**Multimedia Appendix 6.** EB Reference: This intervention includes HF expert-reviewed presence or absence of risk factors listed in HF guidelines and HF expert’s impression on the presence or absence of risk factors as reviewing a surgical case.

**
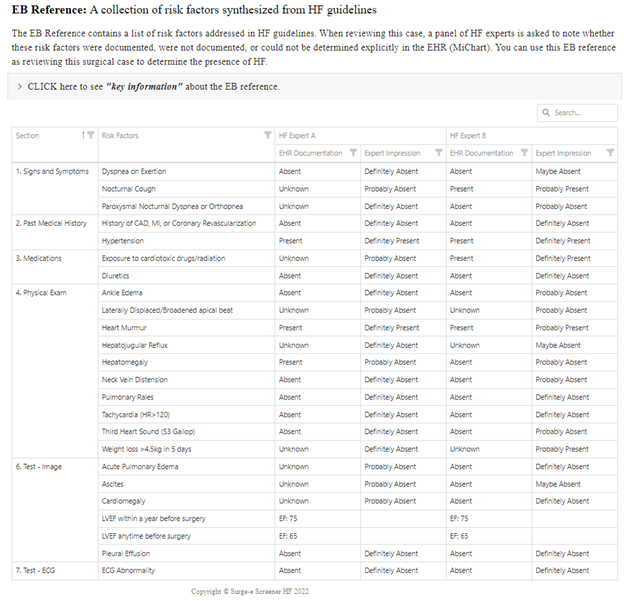
**
