# Supplementary material for: Applying AI and Guidelines to Assist Medical Students in Recognizing Patients With Heart Failure: Protocol for a Randomized Trial
Source: JMIR Res Protoc. 2023 Oct 24;12:e49842. doi: 10.2196/49842 (PMC10630872; doi:10.2196/49842)
Supplement: Multimedia Appendix 7 [file resprot_v12i1e49842_app7.docx]

**Appendix 7.** This is a detailed 7-step procedure to systematically select two sets of surgical cases from electronic health records taking into account factors such as case complexity, expert agreement, machine learning performance, and heart failure outcome.

The selection procedure involved several steps:

**Step 1. Selection of cases from the holdout dataset**

The selection process of surgical cases starts with utilizing a *holdout dataset* that remains unseen during the model training. The 250 cases were randomly chosen for inclusion in the holdout dataset from a pool of 703 expert-adjudicated surgical cases. This step allowed medical students to review new cases that are free from any influence from the model’s prior knowledge.

**Step 2. Agreement among HF experts**

In the second step, we excluded surgical cases with disagreement between HF experts to minimize confusion for students, as the primary objective of this study is HF recognition enhancement in preoperative care for medical students. Following this step, 223 surgical cases were selected after excluding 27 ambiguous surgical cases.

**Step 3. The number of clinical visits**

To ensure adequate EHR data for reviewing surgical cases, a criterion of a minimum number of 10 clinical visits prior to surgery was employed in this selection procedure. This criterion enables medical students to search for and recognize HF risk indicators by reviewing a patient’s medical history and condition from previous clinic visits. In this step, 216 surgical cases remained after removing 7 surgical cases in less than 10 clinic visits.

**Step 4. The easy- and difficult-level surgical cases in HF recognition**

Patients at different stages of HF progression exhibit different levels of recognizing HF. To develop a set of surgical cases with varying difficulty, a stratified approach was adopted considering the agreement between HF experts’ judgment and what is documented in the EHR. For example, easy-level cases were classified when HF ICD codes, LVEF, or HF keywords were documented and aligned with the experts’ judgment, and difficult-level cases were assigned when there was a misalignment between the HF documentation in EHR and experts’ judgment. In this step, 191 cases (93 HF, 98 no HF) were classified as easy-level, and 25 cases (12 HF, 13 no HF) as difficult-level.

**Step 5. Selection Priority**

To establish a comprehensive set of surgical cases for our study, we aimed to include a representative mix of 10 easy and 10 difficult cases, comprising 10 cases with HF and 10 without HF. The surgical cases that were reached to a consensus with a high level of confidence were included in the sets. Furthermore, these selected surgical cases were reviewed by our co-authors to ensure that high-quality surgical cases were included in the study.

**Step 6. ML Prediction Missed**

The performance of our model [39] for this study achieved 90% of AUROC, 82% of sensitivity, and 82% of specificity. To incorporate the performance of the ML model in the final set of 20 surgical cases, we contemplated the inclusion of 16 surgical cases that were accurately predicted by the ML model, as well as 4 surgical cases that were incorrectly detected by the model. Notably, the 4 incorrect surgical cases were difficult-level cases, and no inaccurate prediction from the pool of easy-level surgical cases.

**Step 7. Two Surgical Case Sets for pre- and post-tests**

In this step, the 20 surgical cases were randomly stratified into two sets consisting of 10 cases each, taking into account the level of difficulty, presence or absence of HF, and the ML prediction outcomes. The first set was denoted as Set A, and the second set was denoted as Set B, which were utilized in the pre- and post-tests. As a result, each set includes 10 surgical cases with an equal number of difficult and easy surgical cases, an equivalent distribution of cases with and without HF, and 8 accurately predicted cases and 2 inaccurately predicted cases by the ML model.
