# Supplementary material for: Applying AI and Guidelines to Assist Medical Students in Recognizing Patients With Heart Failure: Protocol for a Randomized Trial
Source: JMIR Res Protoc. 2023 Oct 24;12:e49842. doi: 10.2196/49842 (PMC10630872; doi:10.2196/49842)
Supplement: Multimedia Appendix 8 [file resprot_v12i1e49842_app8.docx]

**Multimedia Appendix 8.** The questionnaire that study participants are asked to answer after reviewing each case via the web-based educational tool.

Question 1. Before the start of this surgery, do you think this patient had CHRONIC HEART FAILURE (i.e., structural heart disease with >3 months of current or prior heart failure symptoms)?

- Yes
- No

Question 2. What is the level of your CONFIDENCE in your prior (Question 1) response?

- Completely Certain (>=95%)
- Very Certain (80-94%)
- Moderately Certain (65-79%)
- Somewhat Certain (50-64%)
- Not at All Certain (<50%)

For cases without intervention (pre-test):

Question 3. Which information INFLUENCED your clinical decision the most for Question 1 while reviewing this case?

- Documentation of a diagnosis of heart failure (or lack thereof)
- Cardiac imaging data (e.g., TTE, cardiac catheterization, etc.) or lack thereof
- Cardiac ECG (or lack thereof)
- Medications (or lack thereof)
- Documented signs and symptoms (or lack thereof)
- Other medical history besides heart failure (or lack thereof)
- Documented physical exam findings / vitals (or lack thereof)
- Laboratory testing (or lack thereof)
- Other (please describe):

For cases with ML_DR_ Intervention (post-test):

Question 3. Which information INFLUENCED your clinical decision the most for Question 1 while reviewing this case?

- HF recommendation by a machine learning algorithm
- Top 5 most important risk factors for heart failure
- Risk factors, Odds Ratio, and Relative Significance in Table
- Documentation of a diagnosis of heart failure (or lack thereof)
- Cardiac imaging data (e.g., TTE, cardiac catheterization, etc.) or lack thereof
- Cardiac ECG (or lack thereof)
- Medications (or lack thereof)
- Documented signs and symptoms (or lack thereof)
- Other medical history besides heart failure (or lack thereof)
- Documented physical exam findings / vitals (or lack thereof)
- Laboratory testing (or lack thereof)
- Other (please describe):

For cases with ML_IR_ Intervention (post-test):

Question 3. Which information INFLUENCED your clinical decision the most for Question 1 while reviewing this case?

- TPR and FPR of a machine learning algorithm
- Top 5 most important risk factors for heart failure
- Risk factors, Odds Ratio, and Relative Significance in Table
- Documentation of a diagnosis of heart failure (or lack thereof)
- Cardiac imaging data (e.g., TTE, cardiac catheterization, etc.) or lack thereof
- Cardiac ECG (or lack thereof)
- Medications (or lack thereof)
- Documented signs and symptoms (or lack thereof)
- Other medical history besides heart failure (or lack thereof)
- Documented physical exam findings / vitals (or lack thereof)
- Laboratory testing (or lack thereof)
- Other (please describe):

For cases with EB Intervention (post-test):

Question 3. Which information INFLUENCED your clinical decision the most for Question 1 while reviewing this case?

- HF experts’ verification (Present, Absent, or Unknown) of risk factors documented in EHR
- HF experts’ impression (Definitely, Probably, or Maybe) of risk factors documented in EHR
- Documentation of a diagnosis of heart failure (or lack thereof)
- Cardiac imaging data (e.g., TTE, cardiac catheterization, etc.) or lack thereof
- Cardiac ECG (or lack thereof)
- Medications (or lack thereof)
- Documented signs and symptoms (or lack thereof)
- Other medical history besides heart failure (or lack thereof)
- Documented physical exam findings / vitals (or lack thereof)
- Laboratory testing (or lack thereof)
- Other (please describe):
