# Supplementary material for: Applying AI and Guidelines to Assist Medical Students in Recognizing Patients With Heart Failure: Protocol for a Randomized Trial
Source: JMIR Res Protoc. 2023 Oct 24;12:e49842. doi: 10.2196/49842 (PMC10630872; doi:10.2196/49842)
Supplement: Multimedia Appendix 9 [file resprot_v12i1e49842_app9.docx]

**Multimedia Appendix 9**. The disclosure of the use of ChatGPT or other generative language models.

ChatGPT was used to assist in writing this manuscript to improve the style of academic writing and grammar checking. The prompt command used in this writing is “rephrase the sentences below in academic writing style: (my writing)” or similar to this prompt command for improving writing style. The author first wrote paragraphs in this manuscript, then used the prompt to rephrase the paragraphs, which had been re-revised by the author to improve the academic writing style before including them in the manuscript.
